# Metabolomic Analysis of Mouse Embryonic Fibroblast Cells in Response to Autophagy Induced by Acute Starvation

**DOI:** 10.1038/srep34075

**Published:** 2016-10-05

**Authors:** Sensen Shen, Rui Weng, Linnan Li, Xinyuan Xu, Yu Bai, Huwei Liu

**Affiliations:** 1 Beijing National Laboratory for Molecular Sciences, Key Laboratory of Bioorganic Chemistry and Molecular Engineering of Ministry of Education, Institute of Analytical Chemistry, College of Chemistry and Molecular Engineering, Peking University, Beijing, 100871, China; 2 Institute of Quality Standard and Testing Technology for Agro-Products, Chinese Academy of Agricultural Sciences, Beijing, 100081, China.

## Abstract

Autophagy-related protein 7 (Atg7) is essential in the formation of the autophagophore and is indispensable for autophagy induction. Autophagy will exist in lower level or even be blocked in cells without Atg7. Even though the possible signaling pathways of Atg7 have been proposed, the metabolomic responses under acute starvation in cells with and without Atg7 have not been elucidated. This study therefore was designed and aimed to reveal the metabolomics of Atg7-dependent autophagy through metabolomic analysis of Atg7^*−/−*^ mouse embryonic fibroblast cells (MEFs) and wild-type MEFs along with the starvation time. 30 significantly altered metabolites were identified in response to nutrient stress, which were mainly associated with amino acid, energy, carbohydrate, and lipid metabolism. For the wild-type MEFs, the induction of autophagy protected cell survival with some up-regulated lipids during the first two hours’ starvation, while the subsequent apoptosis resulted in the decrease of cell viability after four hours’ starvation. For the Atg7^*−/−*^ MEFs, apoptosis perhaps led to the deactivation of tricarboxylic acid (TCA) cycle due to the lack of autophagy, which resulted in the immediate drop of cellular viability under starvation. These results contributed to the metabolomic study and provided new insights into the mechanism associated with Atg7-dependent autophagy.

Autophagy, the adaptive degradation process of cytoplasmic constituents in the lysosome/vacuole under stress or poor nutrient conditions, is essential for the maintenance of metabolic homeostasis and viability of cells^1^,^2^. It is highly conserved from yeast to mammals, both morphologically and with regard to the protein constituents that make up the core autophagy machinery. Through autophagy, damaged cells or organelles will be eliminated or degraded to provide the necessary nutrients for its repair, reconstruction or regeneration. It is widely believed that autophagy is a defensive stress mechanism, and can be regarded as the recycling center^3^.

It has been reported that autophagy is tightly connected with the cellular development and differentiation, and various human diseases including cancers, neurodegenerative disorders, aging, or innate and adaptive immunity^4^,^5^. The pro-survival functions of autophagy have been demonstrated at the organismal level and cellular level under different conditions, for example nutrient and growth factor deprivation and microbial infection etc.^6^,^7^,^8^,^9^. However, more and more researches proved that autophagy can act both in cytoprotection and in cell death, that is autophagy can prevent various diseases with a cytoprotective mechanism while the dysfunctional autophagy leads to pathology^10^. Thus better understanding of autophagy is of great significance to find new ways to prevent or treat diseases.

Autophagy-related (Atg) genes, which encode the autophagy-related proteins, are key regulators of autophagy which control its induction and the formation of autophagophore under stress or poor nuturient conditions^11^. Since the discovery of Atg genes and Atg proteins, the prelude of the study of autophagy in molecular level was opened. Among the key Atg proteins, Atg7, which is homologous to the ubiquitin-activating enzyme E1 (Uba1), encodes the E1-like enzyme that is essential to these conjugation systems and is indispensable for both selective and nonselective autophagy induction^12^. Autophagy will exist in lower level or even be blocked in Atg-deficient cells^13^ or mice^14^. Previous studies have revealed that Atg7 is essential for normal lifespan and tolerance to starvation and oxidation in cell lines^15^,^16^, *C. elegans* and *Drosophila* models^17^. Atg7-dependent autophagy is required for acrosome biogenesis during spermatogenesis in mice^18^. Besides, some researchers reported that suppression of basal autophagy in neural cells can cause neurodegenerative disease in mice^19^,^20^.

Metabolomics, an emerging ‘-omics’ approach of system biology, is the downstream product of genomics, transcriptomics and proteomics that can often provide unexpected and unique insights into various biological processes^21^. Since changes in the metabolome represent the final response of an organism to both internal and external stimuli, metabolomics is particularly conducive to identifying pathophysiologically affected processes and moreover elucidating novel physiological and pathological mechanisms^22^. One recent publication focused on the metabolic regulation of autophagy^23^, while the effects of autophagy on metabolism have rarely been studied.

In the present study, an ultra-performance liquid chromatography-Q-Exactive mass spectrometry (UPLC-MS) method was employed to conduct metabolomic analysis of Atg7-deficient (Atg7^*−/−*^) mouse embryonic fibroblast cells (MEFs) *vs* wild-type MEFs along with the starvation time. Through identifying the metabolic differences of these two cell lines in response to acute starvation, the metabolomic network and related pathways associated with Atg7-dependent autophagy were proposed, which provided fundamental data for the molecular mechanism and gave new insights into the understanding of autophagy.

## Results

### Morphology characteristics and cell viability

**Figure 1:**
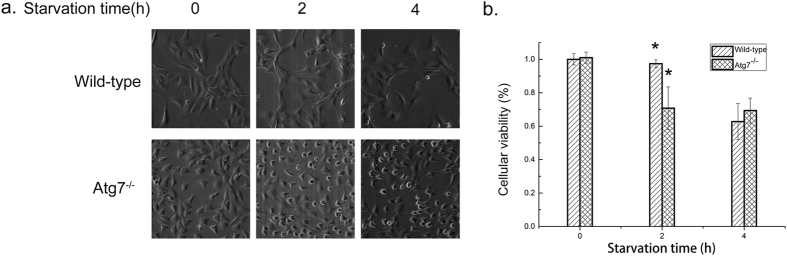
Morphology characteristics and cellular viability results. (**a)** Morphology characteristics of wild-type and Atg7^*−/−*^ MEFs under different starvation time. (**b)** Effects of acute starvation on cellular viability of wild-type and Atg7^*−/−*^ MEFs (n = 3, *p < 0.05).

When cultured in the normal medium, Atg7^*−/−*^ MEFs grew faster than wild-type MEFs. However, after exposed to EBSS for 2 hours, Atg7^*−/−*^ MEFs showed an obvious phenomenon of cell death while wild-type MEFs did not (Fig. 1a). And after starvation for 4 hours, about 40% cells died in Atg7^*−/−*^ MEFs as well as in wild-type MEFs. The results of cellular viability test were consistent with the changes of morphology characteristics (Fig. 1b). The cellular viability of wild-type MEFs did not change a lot until starvation for 4 hours, while cellular viability of Atg7^*−/−*^MEFs decreased very fast at the first 2 hours then kept stable in the next two hours’ starvation. These results revealed the different responses to acute starvation in the two cell lines especially in the first period of starvation.

### Global metabonomic analysis and altered metabolites identification

**Figure 2:**
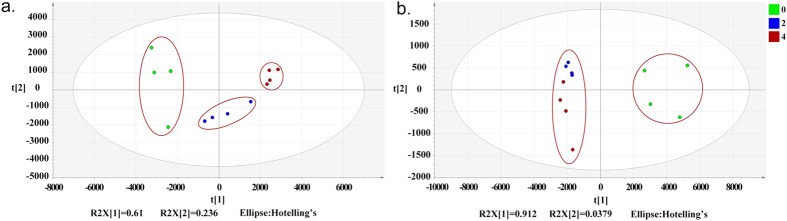
Multivariate statistical analysis based on the metabolomic profiling data of MEFs under different starvation time. (**a)** PCA scores plot of wild-type MEFs (R2X[1] = 0.610, R2X[2] = 0.236). (**b)** PCA scores plot of Atg7^*−/−*^ MEFs (R2X[1] = 0.912, R2X[2] = 0.0379). Green: 0 hour, Blue: 2 hours, Red: 4 hours.

A UPLC-MS analytical method was developed to obtain and identify potential metabolites perturbed by the acute starvation stimuli in the wild-type MEFs and Atg7^*−/−*^ MEFs. PCA score was employed to get the overview of differences between different groups based on the metabolomic profiling data. For wild-type MEFs, PCA demonstrated clear differences between cells under different starvation time (Fig. 2a). While for Atg7^*−/−*^ MEFs, cells starved for 2 hours and 4 hours cannot be distinguished very clearly (Fig. 2b). This indicated that the knockout of Atg7 gene indeed affected the metabolomic responses to acute starvation.

**Figure 3:**
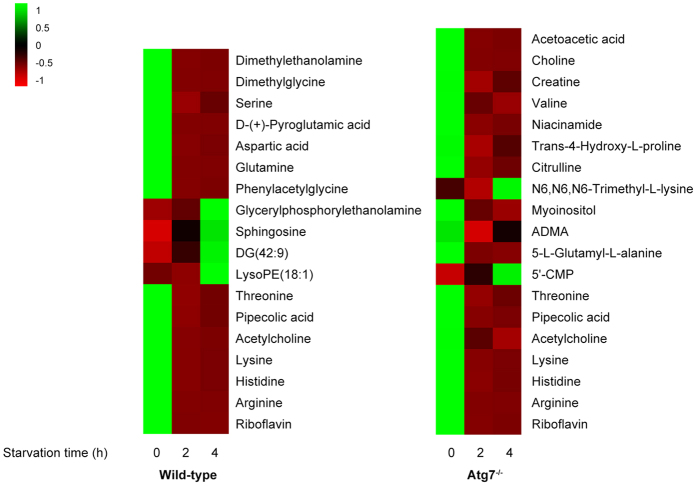
Heat maps denoting the fold change of the altered metabolites in wild-type MEFs and Atg7^*−/−*^ MEFs. Columns correspond to cell groups under different starvation time, and rows correspond to different altered metabolites. Shades of green represent elevated levels of content, while shades of red represent reduced levels of content.

In total, 156 ions of wild-type MEFs and 180 ions of Atg7^*−/−*^ MEFs were found to meet the selection criteria (*P-*value < 0.05, max fold change >2). After excluding ions with intensity <10^5^, we finally selected 57 ions (wild-type MEFs) and 76 ions (Atg7^*−/−*^ MEFs) respectively which were significantly altered during the starvation time. 18 metabolites in wild-type MEFs and 19 metabolites in Atg7^*−/−*^ MEFs were finally identified, among which 7 metabolites were overlapped (Table 1). The change tendencies of these metabolites were shown in the form of heat map (Fig. 3), based on the averaged peak area of each metabolite. They were mainly amino acids, lipids and their precursors or related derivatives. In wild-type MEFs, glycerylphosphorylethanolamine, sphingosine (d18:1), DG(42:9) and LysoPE(18:1) had an increasing tendency during starvation. While in Atg7^*−/−*^ MEFs, only N6,N6,N6-Trimethyl-L-Lysine and 5′-CMP were increasing while other metabolites were all significantly decreasing. Besides, the overlapped 7 metabolites were detected significantly down-regulated along with the starvation time (Fig. 3), which implied that some physiological processes these metabolites involved in were induced in both of the two cell lines. However, there are still many other differentiated metabolites that were unknown so far, we will attempt to identify them in our future studies (Supplementary Tables S1 and S2).

### Metabolic pathway analysis

For the identified metabolites of wild-type MEFs and Atg7^*−/−*^ MEFs, metabolic pathway analysis was conducted using MetaboAnalyst 3.0. In wild-type MEFs, 19 metabolic pathways were disturbed which suggested the perturbation of amino acid metabolism, energy metabolism, lipid metabolism, nucleotide metabolism and others (Supplementary Fig. S1, Supplementary Table S3). In Atg7^*−/−*^ MEFs, 22 metabolic pathways were perturbed mainly belonging to energy metabolism, amino acid metabolism, carbohydrate metabolism (Supplementary Fig. S2, Supplementary Table S3). After summarizing the specific pathways of the two cell lines, some disturbed metabolic pathways are the same, while others are different (Supplementary Table S3). Glycine, serine and threonine metabolism, arginine and proline metabolism, lysine degradation and biosynthesis, histidine metabolism, nitrogen metabolism and riboflavin metabolism were all disturbed in both of the two cell lines. Some different altered pathways mainly referred to the amino acid metabolism, energy metabolism, lipid metabolism, carbohydrate metabolism and metabolism of cofactors and vitamins.

## Discussion

**Figure 4:**
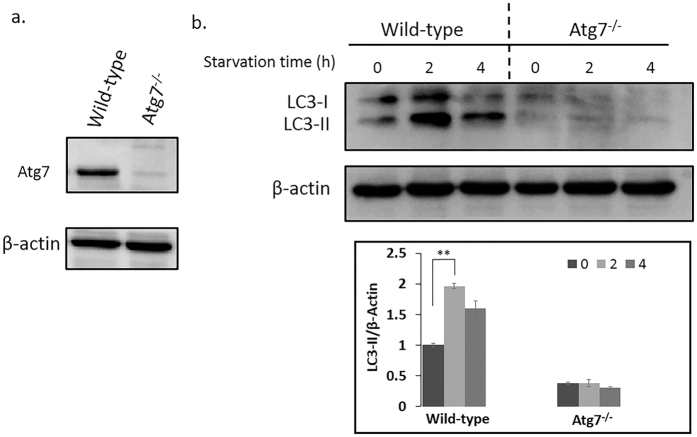
Western blot results of Atg7 and the conversion of LC3-I to LC3-II along with the starvation time. (**a)** Levels of Atg7 were confirmed in wild-type and Atg7^*−/−*^ MEFs. (**b)** Levels of LC3-II were used to compare autophagy induced by acute starvation in wild-type MEFs and Atg7^*−/−*^ MEFs. Cells were treated with EBSS for the indicated times, and protein was extracted and analyzed for Atg7, LC3-I and LC3-II. Error bars indicate the SD (n = 3), **P < 0.01.

Mammalian autophagy can be induced by a variety of nutrient stresses and other cellular insults through signaling pathways, for example the glucose deprivation, amino acid withdrawal, mitochondria damage and so on. Induced autophagy can provide nutrients for mitochondrial oxidation, suppress p53 pathway activation, and ultimately delay apoptosis^24^. Atg7, which encodes the Uba1-like enzyme, is essential in the formation of the autophagophore and is indispensable for autophagy induction. Autophagy will exhibit lower levels or even be blocked in the cells without Atg7 gene^16^,^17^,^18^. During the formation of autophagosome, LC3-I will be modified into the PE-conjugated form, LC3-II, which can be regarded as the marker of autophagy. Atg8—PE/LC3-II is the only protein marker that is reliably associated with completed autophagosomes^25^. In our study, the level of Atg7 in wild-type and Atg7^*−/−*^ MEFs was confirmed by western blot (Fig. 4a), which proved the successful knockout of Atg7. Besides, based on the quantitative western blot results of LC3-I and LC3-II, the degree of the autophagy in wild-type MEFs was significantly elevated during the first two hours of acute starvation then slightly decreased (Fig. 4b). However, in the Atg7^*−/−*^ MEFs, the autophagy was inhibited (Fig. 4b).

**Figure 5:**
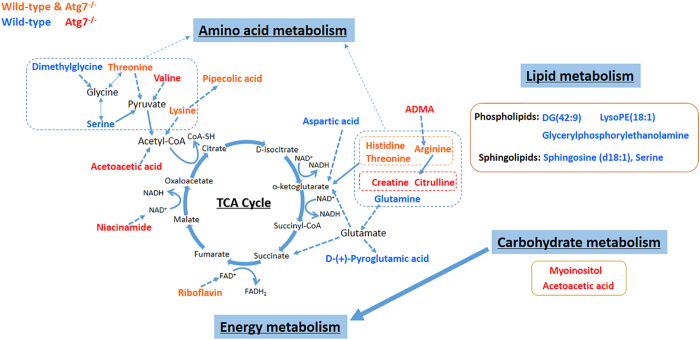
An overview of the integrated metabolic pathway network of wild-type MEFs and Atg7^*−/−*^ MEFs in response to acute starvation. Red-labeled metabolites are identified in Atg7^*−/−*^ MEFs, blue-labeled metabolites are identified in wild-type MEFs. Orange-labeled metabolites are intersection of these compounds. Metabolite relationships were derived from HMDB and KEGG databases. Solid arrows represent direct metabolic reactions, and dashed arrows represent multiple reactions and indirect connections between two metabolites.

Based on the overall identified altered metabolites in wild-type MEFs and Atg7^*−/−*^ MEFs during the starvation time, metabolic pathway analysis suggested the same and different perturbed pathways in the two cell lines, which implied that after the knockout of Atg7, the metabolic responses of MEFs to acute starvation changed. Combining the pathways and altered metabolites, a correlated pathway network was constructed as shown in Fig. 5.

MetPA analysis indicated that amino acid metabolic pathways were affected the most due to the acute starvation (Supplementary Table S3, Fig. 5). In wild-type MEFs, the altered metabolic pathways were mainly D-glutamine and D-glutamate metabolism, glycine, serine and threonine metabolism, histidine metabolism and alanine, aspartate and glutamate metabolism. However, in Atg7^*−/−*^ MEFs, mainly arginine and proline metabolism, histidine metabolism and lysine degradation were disturbed. Besides, Alanine, aspartate and glutamate metabolism, cysteine and methionine metabolism, and phenylalanine metabolism were only dysregulated in wild-type MEFs. Valine, leucine and isoleucine biosynthesis and degradation were only disturbed in Atg7^*−/−*^ MEFs as well as tyrosine metabolism. Especially, aminoacyl-tRNA biosynthesis was also perturbed in both of the cell lines reflecting the disturbance of amino acid metabolism from the genetic translation process.

It has been reported that depletion of amino acids can trigger autophagy through various mechanisms, for example activating transcription factor 4^26^, inhibition of proline hydroxylases^27^ and so on. But they mainly laid emphasis on the metabolic triggers and the proteomic pathways through which they were involved in the autophagy such as the AMPK^28^,^29^ or mTORC1^30^,^31^,^32^. Our research revealed the effects of acute starvation-induced autophagy on amino acid metabolism from the opposite angle and revealed the close connection between them.

TCA cycle is a quite important process to generate energy in the form of adenosine triphosphate (ATP) through the oxidation of acetate into carbon dioxide and reducing NAD^+^ to NADH^33^. The overview of the TCA cycle is shown in Fig. 5, with some important intermediates and the detected metabolites in our study. Briefly, acetyl coenzyme A (acetyl-CoA) is an important molecule in TCA cycle which can convey the carbon atoms within the acetyl group to be oxidized^34^. We found that threonine, lysine, pipecolic acid were decreasing in both of the two cell lines (Fig. 3). Serine significantly decreased under two hours’ starvation then slightly increased in wild-type MEFs (Fig. 3). Valine was down-regulated in Atg7^*−/−*^ MEFs as well as acetoacetic acid (Fig. 3). These implied the disturbance of metabolic pathways related with TCA cycle (Fig. 5). Pipecolic acid was a metabolite of lysine, thus they have the same variation tendency. Valine was involved in the pathways of pantothenate and CoA biosynthesis, which can convert into pyruvate. Threonine, serine and lysine can be converted into acetyl-CoA through oxidation of pyruvate or other metabolic reactions (Fig. 5).

**Figure 6:**
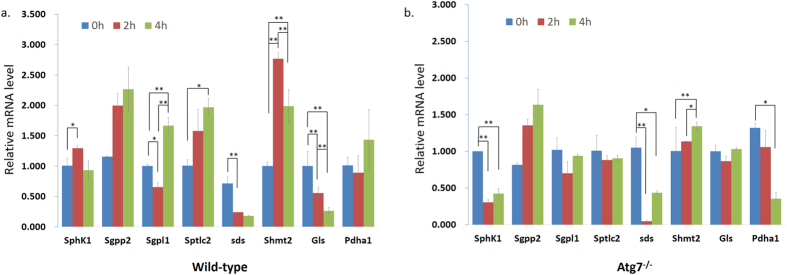
Expression of related proteins along with the starvation time. (**a)** The related enzymes mRNA expression in wild-type MEFs and (**b)** the related enzymes mRNA expression in Atg7^*−/−*^ MEFs under different starvation time. RNA was extracted and analyzed by quantitative PCR. Cells were treated with EBSS for the indicated times. Error bars indicate the SD (n = 3). *p < 0.05; **p < 0.01.

To further confirm our metabolic pathway hypothesis, the qPCR analysis of cells under different starvation time were conducted which revealed that starvation dramatically changed the mRNA level of some important related genes belonging to different altered pathways (Fig. 6). We took into account the results of MetPA (Supplementary Table S3) and the identified metabolites related with autophagy to choose those genes based on KEGG (Supplementary Table S5) to explain or validate the altered metabolites and disturbed pathways.

Among them, Sds and Shmt2 belong to ‘Glycine, serine and threonine metabolism’ pathway, which was one of the most affected pathways according to the MetPA analysis. Starvation induced the up-regulation of Shmt2, which encodes an enzyme that can catalyze reactions between serine and glycine, in wild-type MEFs under the first two hours’ starvation, then down-regulated afterwards. This implied that the convert from serine to glycine increased firstly then decreased gradually. However the mRNA level of Sds, encoding a very important enzyme that converts serine to pyruvate, was dramatically decreasing under starvation in both of the cell lines (Fig. 6). These together with the decreasing threonine, valine, lysine and acetoacetic acid indicated the synthesis of pyruvate was inhibited which will definitely affect the normal supplement of acetyl-CoA needed by the TCA cycle.

α-ketoglutarate is also a key intermediate in the TCA cycle, which can be produced by the oxidative deamination of glutamate by glutamate dehydrogenase^35^. In wild-type MEFs, glutamine was detected to be significantly down-regulated along with the starvation time (Fig. 3). Since glutamine can be converted into glutamate through glutaminase (Gls)^35^, its decreasing reduced the content of glutamate then α-ketoglutarate subsequently (Fig. 5). Furthermore, the mRNA level of Gls was dramatically decreasing under starvation in wild-type MEFs while quite stable in Atg7^*−/−*^ MEFs (Fig. 6), which also proved the inhibition of synthesis of glutamate in wild-type MEFs. D-(+)-pyroglutamic acid, the cyclic derivative of glutamate, was also down-regulated (Fig. 3), which was just consistent with the trend of glutamine.

In addition, histidine, arginine and threonine were all decreasing in both of the two cell lines (Fig. 3). Threonine can be converted into α-ketobutyrate in a less common pathway via the enzyme serine dehydratase, except for the conversion to pyruvate via threonine dehydrogenase, and thereby enter the pathway leading to succinyl-CoA. Histidine can also transform into α-ketobutyrate as well as arginine (Fig. 5). The down-regulation of the three amino acids will also affect the normal level of intermediates in TCA cycle. Moreover, citrulline, creatine, ADMA (Asymmetric dimethylarginine) and valine were significantly decreasing in Atg7^*−/−*^ MEFs (Fig. 3). That is because citrulline can be derived from arginine via nitric oxide synthase and arginine is necessary for the synthesis of creatine^36^. ADMA is also closely related with arginine^37^, thus its decreasing can be explained (Fig. 5).

Besides, niacinamide was down-regulated in Atg7^*−/−*^ MEFs and riboflavin was decreasing in both of the two cell lines during starvation (Fig. 3). They were involved in the metabolism of cofactors and vitamins. Niacinamide is an important compound functioning as a component of the coenzyme NAD^38^, and riboflavin is the central component of the cofactors FAD^39^ (Fig. 5). NAD and FAD are required in the TCA cycle, with the reduction of NAD to NADH and FAD to FADH_2_ respectively. The NADH generated in the TCA cycle may later donate its electrons in oxidative phosphorylation to drive ATP synthesis and FADH_2_ is covalently attached to succinate dehydrogenase, acting as an intermediate in the electron transport chain. Thus the down-regulation of niacinamide and riboflavin will deactivate the TCA cycle as well.

Carbohydrate metabolism pathways were affected only in the Atg7^*−/−*^ MEFs, reflected through the significantly decreasing of acetoacetic acid and myoinositol (Fig. 3). Carbohydrate metabolism is quite close with TCA cycle, since pyruvate can be synthesized from glycolysis, and is also a fundamental biochemical process to ensure a constant energy supply of living cells. Acetoacetic acid was involved in the butanoate metabolism, in which it can be converted into acetyl-CoA via steps of reactions, thus its decreasing was also indicating the deactivation of TCA cycle (Fig. 5). And this indicated that the inhibition of TCA cycle in Atg7^*−/−*^ MEFs was stronger than that of wild-type MEFs, which can also be indicated by the down-regulated mRNA level of Pdha1 in Atg7^*−/−*^ MEFs instead of wild-type MEFs (Fig. 6), since Pdha1 encodes an enzyme that catalyzes the irreversible oxidative decarboxylation of pyruvate to produce acetyl-CoA in the bridging step between glycolysis and the citric acid cycle. Furthermore, myoinositol was involved in inositol phosphate metabolism and galactose metabolism, which is produced from glucose. It can serve as an important component of the structural lipids phosphatidylinositol (PI) and its various phosphates—the phosphatidylinositol phosphate (PIP) lipids^40^,^41^. Articles have reported that myoinositol and its polyphosphates, functioning as the secondary messenger, are involved in many biological processes including insulin signal transduction^42^, cell membrane potential maintain^43^ and so on. Thus, the down-regulation of myoinositol implied the abnormity of signal transduction in Atg7^*−/−*^ MEFs except for the deactivation of TCA cycle.

Lipid metabolism was greatly affected mainly in the wild-type MEFs. DG(42:9), LysoPE(18:1) and glycerylphosphorylethanolamine were up-regulated along with the starvation time as well as the sphingosine, which forms a primary part of sphingolipids (Fig. 3). Since autophagy was induced in wild-type MEFs and the autophagosome is a membrane structure, the increase of lipid metabolism can be explained. The mRNA level of Sptlc2 was up-regulated in wild-type MEFs while not in Atg7^*−/−*^ MEFs (Fig. 6), which also implied that the production of sphingoid bases from amino acids was enhanced. To better interpret the lipid metabolism related to the Atg7-dependent autophagy, a more comprehensive and specific lipidomic analysis is currently underway to find out more kinds of lipid species and pathways associated with this biological process.

**Figure 7:**
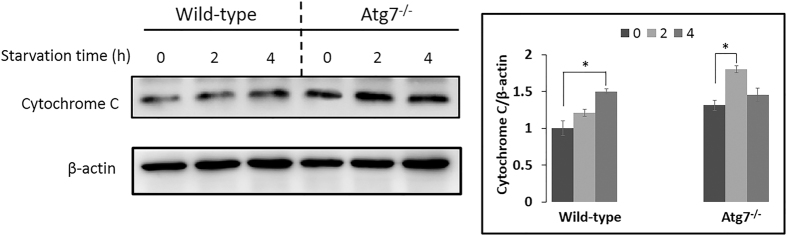
Western blot results of the cytochrome C along with the starvation time. Wild-type MEFs and Atg7^*−/−*^ MEFs were treated with EBSS for the indicated times and protein was extracted and analyzed for cytochrome C. Error bars indicate the SD (n = 3), *p < 0.05.

Especially, sphingosine (d18:1) was detected to be significantly increasing (Fig. 3). Sphingolipid metabolites, such as sphingosine and sphingosine-1-phosphate (S1P), are lipid signaling molecules involved in diverse cellular processes, which can be mutual transformed (Supplementary Fig. S3). Sphingosine (d18:1) was reported to be a positive regulator of apoptosis^44^ while S1P can induce autophagy protecting from cell death with apoptotic hallmarks^45^. But our results revealed that the sphingosine (d18:1) was increasing even though autophagy was induced (Figs 3 and 4). Further western blot results proved the occuring of apoptosis along with the starvation time (Fig. 7), and based on the quantitative results of the intensity, apoptosis in Atg7^*−/−*^ MEFs was much stronger compared with wild-type MEFs regardless of starvation, which indicated Atg7^*−/−*^ MEFs are perhaps more sensitive to apoptosis under normal condition. Besides, the degree of apoptosis did not changed significantly during first two hours’ starvation in wild-type MEFs and significantly increased till four hours’ starvation. However, apoptosis was significantly induced under two hours’ starvation then slightly decreased in Atg7^*−/−*^ MEFs (Fig. 7). These results were consistent with the cellular viability results shown in Fig. 1. Then the same m/z of sphingosine (d18:1) in the Atg7^*−/−*^ MEFs was extracted at different starvation time and get the averaged peak area of each group. The content of sphingosine (d18:1) and the rate of increasing were greater in Atg7^*−/−*^ MEFs than in wild-type MEFs under two hours’ starvation (Supplementary Fig. S4). This further confirmed our assumption that during the first period of starvation, the influence of apoptosis was much greater in Atg7^*−/−*^ MEFs than wild-type MEFs, due to the lack of autophagy, while autophagy was the dominant process in the latter.

These results can also be interpreted from the mRNA level of Sphk1, Sgpp2 and Sgpl1 which belong to ‘Sphingolipid metabolism’ pathway and will affect the balance of mutual transformation between sphingosine and S1P (Supplementary Table S5, Supplementary Fig. S3). The first two hours’ starvation induced the up-regulation of Sphk1 in wild-type MEFs while dramatic down-regulation in Atg7^*−/−*^ MEFs. Meanwhile, the Sgpp2 mRNA was up-regulated in both of the two cell lines (Fig. 6). These implied that the balance between sphingosine and S1P was quite stable in wild-type MEFs, but in Atg7^*−/−*^ MEFs, the balance was destroyed with S1P greatly converted into sphingosine. As the starvation time prolonged, Sphk1 mRNA went down back to the normal level in wild-type MEFs, while no obvious change in Atg7^*−/−*^ MEFs (Fig. 6). This caused the increase of sphingosine in wild-type MEFs and the stable level in Atg7^*−/−*^ MEFs in the next two hours’ starvation, since the Sgpp2 mRNA had little change in the two cell lines. In addition, the mRNA level of Sgpl1 was quite stable in Atg7^*−/−*^ MEFs but it increased in wild-type MEFs after four hours’ starvation (Fig. 6), which indicated the greater consumption of S1P as well as the increase of apoptosis. These together implied that acute starvation did not break the balance between sphingosine and S1P in wild-type MEFS obviously due to autophagy, which delayed cell death, in the first two hours’ starvation. However, in Atg7^*−/−*^ MEFs, sphingosine dramatically increased under starvation due to apoptosis instead of autophagy, which caused the immediate cell death. And this perhaps caused the different metabolic responses to acute starvation in the two cell lines subsequently.

The relationship between autophagy and apoptosis is complex. Autophagy can also lead to cell death, possibly through activating apoptosis^46^ or possibly as a result of the inability of cells to survive the non-specific degradation of large amounts of cytoplasmic contents^47^. The crosstalk between autophagy and apoptosis is complicated since the two pathways shared some common factors^9^. Some efforts have been made to figure out the intricate interaction between them^48^,^49^,^50^,^51^, but only get tip of the iceberg. Our results revealed the close connection between autophagy and apoptosis from the metabolomics perspective. Thus, cellular viability was decreasing under two hours’ starvation in Atg7^*−/−*^ MEFs since only apoptosis was greatly induced, while kept stable in wild-type MEFs in the first two hours’ starvation due to the dominant process of autophagy. However, with the longer duration of starvation, apoptosis was enhanced with the reduced degree of autophagy, cell viability was decreasing gradually in wild-type MEFs (Figs 1, 4 and 7). We also added a recovery group (R), in which cells were re-cultured in the full medium after 4 hours’ starvation, as the control group to confirm these results. Results were shown in Supplementary Fig. S5, which were consistent with Figs 4 and 7. And even though re-cultured in the full medium, the cytochrome C release in Atg7^*−/−*^ MEFs still in higher level compared to wild-type MEFs just as under 0 hour starvation.

In summary, we used a UPLC-MS method to identify significantly altered metabolites of MEFs with and without Atg7 in response to the acute starvation and to elucidate the affected metabolic pathways for the first time. Nutrient starvation induced autophagy in wild-type MEFs with up-regulated lipid metabolism and it was dominant prior to the apoptosis, which delayed cell death. But under four hours’ starvation, the degree of apoptosis increased with the decrease of autophagy affecting the amino acids metabolism, carbohydrate metabolism and energy metabolism, which suggested the deactivation of TCA cycle as well as in Atg7^*−/−*^ MEFs, where only apoptosis was occurring. These results proved the necessary of Atg7 in response to acute starvation for survival of MEFs, which opened a new sight on the understanding of the Atg7-depended autophagy, and further studies should focus on the elucidation of the mechanism of Atg7-dependent autophagy in relation to these perturbed metabolic pathways.

## Methods

### Chemicals and reagents

Formic acid (HPLC grade) was purchased from Dikma Technologies Inc. (Lake Forest, CA, USA). Water (HPLC grade) was obtained from Fisher Scientific (Geel, Belgium). Acetonitrile (HPLC grade) was purchased from Sigma-Aldrich (St. Louis, MO, USA). Dulbeco’s modified Eagle’s medium (DMEM) was from Hyclone (Beijing, China). Fetal Bovin serum and GluMAX were from Life Technologies Corporation (Grand Island, NY, USA). Trypsin-EDTA (0.25%) was from Macgene (Beijing, China), and Pbs was from Solarbi science & Technology Co. Ltd. (Beijing, China). Earle’s Balanced Salt Solution (EBSS) was from Sigma-Aldrich (St. Louis, MO, USA). MTT Assay was from Solarbio life sciences (Beijing, China). Antibodies were purchased as follows: rabbit anti-LC3 (Cell Signaling Technology, 3868S), mouse anti-beta-Actin (TransGene Biotech, A005), Anti-rabbit IgG, HRP-linked Antibody (Cell Signaling Technology, 7074S), Goat anti-mouse IgG-HRP (Abmart, M21001L). TRIzol was from life technologies (USA), PrimerScript^TM^ RT reagent Kit with gDNA eraser was from TaKaRa (USA). Ultra SYBR Mixture (with ROX I) was from CWBIO (Beijing, China).

### Cell culture experiments and sample preparation

Wild-type and Atg7^*−/−*^MEF cell lines were cultured in DMEM supplemented with 10% FBS and 1% (v/v) penicillin/streptomycin at 37 °C in an atmosphere of 5% CO_2_. The medium was changed every 24 hours.

The wild-type and Atg7^*−/−*^MEFs were divided into three groups separately. After exposed to EBSS for 0 hour, 2 hours and 4 hours, cells were washed by cold PBS, then cold water and cold methanol (1.5 ml) subsequently. Then they were scraped, homogenized and transferred into 1.5 ml tubes separately. The intracellular metabolism was then rapidly quenched by liquid nitrogen and left at −20 °C for 30 minutes for further metabolites extraction. Finally, they were vortexed and centrifuged at 14000 rpm for 10 minutes and supernatants were taken for further analysis or stored in −80 °C. Each group has four dishes of cells as replicates at each starvation time point, resulting in 6 groups and 24 samples in total.

### UPLC-Q-Exactive-MS Analysis

A quality control (QC) sample was prepared by mixing equal volumes (10 μL) from each sample. This “pooled” sample was used to estimate a “mean” profile representing all the analytes encountered during analysis^52^.

UPLC analysis was performed on an ACQUITY UPLC HSS T3 column (100 mm × 2.1 mm, 1.8 μm; Waters Corp., Dublin, Ireland) using an ACQUITY UPLC^TM^ I-Class (Waters Corp., Milford, USA). A 5 μL injection was made onto the column, which was maintained at 30 °C and eluted with A) water (0.1% (v/v) formic acid, 2% (v/v) acetonitrile) and B) acetonitrile (0.1% (v/v) formic acid) at a flow rate of 0.3 mL/min for 20 min. The gradient duration program was as follows: 0–3 min, 0% B; 3–7 min, 0–40% B; 7–12 min, 40–100% B; 12–18 min, 100% B and re-equilibrated with 0% B for 2 min.

MS analysis was conducted using a Thermo Q-Exactive mass spectrometry (Thermo scientific, USA) operating in positive (HESI+) electrospray ionization mode. The instrument was calibrated using external standard before analysis to ensure the mass accuracy within 3 ppm. The parameters were set as follows: spray voltage was 3.5 KV, capillary temperature was 320 °C, sheath gas flow rate was 30, aux gas flow rate was 10, sweep gas flow rate was 5, the heater temperature was 350 °C, and the S-Lens RF level was 55. Full mass range (m/z 70–1000) with resolution of 70000 was used. MS/MS scan used normalized collision energy of 35 V, an isolation window of 0.8 m/z and a mass resolution of 35000.

The pooled “QC” sample was injected three times at the beginning of the analysis batch to ensure system equilibrium and then every 6 samples to further monitor the analysis stability^53^,^54^. All samples were injected randomly in the batch.

### Data processing

The data were analyzed using Waters Progenesis QI. Raw data were aligned, normalized, deconvoluted and assembled into a data matrix automatically. Data were aligned with the mass tolerance of 5 ppm and a retention time window tolerance of 0.1 min. The data matrix was analyzed by ANOVA and only metabolites with *p*-values less than 0.05 were considered to be statistically significant. Fold changes were calculated from the arithmetic mean values of each group and metabolites with max fold change large than 2 are selected subsequently. Then the data were analyzed by SIMCA to get the principal component analysis (PCA) score. And selected metabolites were further analyzed to be identified.

### Metabolite identification

Selected metabolites were initially compared with comprehensive online databases (METLIN: http://metlin.scripps.edu, mzCloud: http://www.mzcloud.org, HMDB: http://www.hmdb.ca, KEGG: http://www.kegg.com, LIPIDMAPS: http://www.lipidmaps.org and MassBank: http://www.massbank.jp) using exact m/z values and MS/MS fragments. The identification information was then confirmed by chemical standards with the exact m/z values, MS/MS fragments and retention times.

### Metabolic pathway analysis (MetPA) by MetaboAnalyst

Metabolic Pathway Analysis (MetPA) by MetaboAnalyst 3.0 (www.metaboanalyst.ca/) was used to sort the significantly altered metabolites associated with the biological processes along with the starvation time in the wild-type MEFs and Atg7^*−/−*^ MEFs respectively. MetPA, a web-based application that combines pathway enrichment analysis and network topological analysis. It includes metabolic pathways encompassing 21 model organisms and can aid in the visualization of metabolomics data within the biological context of metabolic pathways.

### Cell viability

Cell viability was measured by using 3-(4,5)-dimethylthiahiazo-(z-y1)-3,5-di-phenytetrazoli-umromide (MTT) assay. About 3000 cells were plated in each well of 96-well plates at 37 °C in a humidified 5% CO_2_ for about 12 h before being treated with the indicated compounds. After treatment, 90 μl new culture medium and 10 μl MTT (5 mg/ml) solution was added to each well, and the cultures incubated for 4 h at 37 °C in humidified 5% CO_2_. The medium was extracted to stop the reaction, then 110 μl dimethylsulfoxide (DMSO) was added and measured with a Multiskan MK3 spectrophotometer (Thermo Fisher Scientific, USA) at 490 nm and also at 620 nm as the background to be extracted. Each sample was repeated 3 times for standard deviation calculations.

### Western blot analysis

Cells were first treated with lysis buffer (50 mM Tris, 150 mM NaCl, 1% Triton X-100, 0.5% Sodium deoxycholate, 0.1% SDS, cOmplete Protease Inhibitor, pH 7.4,). Following heat denaturation for 10 min, 20 microgram of proteins were separated by 15% SDS-PAGE and transferred to a 0.22 um nitrocellulose filter. The filter was blocked with 5% BSA in TBST buffer and incubated with the indicated antibodies, then analyzed with enhanced chemiluminescence.

### Real-time quantitative PCR

Cells under different starvation time were lysed and RNA was extracted using TRIzol (Life technologies). cDNA was generated using 1 μg of total RNA by reverse transcription using a cDNA synthesis kit (TaKaRa, RR047A) following the protocol provided. Real-time quantitative PCR (qPCR) was performed on a real-time quantitative PCR system ABI 7500 System (Applied Biosystems, USA). Primes were designed using Primer blast on the National Center for Biotechnology Information website (Supplementary Table S4) and functions of these selected proteins are also summarized (Supplementary Table S5). 1 μl of cDNA was used and primers (Supplementary Table S4) were added to the PCR reaction. All other components for the PCR reaction were from SYBR Mixture kit. The reaction was performed in a volume of 10 μl in triplicate, according to the manufacturer’s instructions.

The relative differences in RNA expression in samples with different starvation time were assessed by the comparative Cт (ΔΔCт) method. Briefly, Cт values were first normalized to that of ACTB (β-actin) in the same sample (ΔCт) and then differences of Cт values between each treated and control group (ΔΔCт) were used to calculated the changes of fold induction in each sample using the formula 2^−ΔΔCт. All qPCR experiments were done in triplicate and the results were averaged.

## Additional Information

**How to cite this article**: Shen, S. *et al*. Metabolomic Analysis of Mouse Embryonic Fibroblast Cells in Response to Autophagy Induced by Acute Starvation. *Sci. Rep.* **6**, 34075; doi: 10.1038/srep34075 (2016).

## Supplementary Material

Supplementary Information

## Figures and Tables

**Table 1:** Altered metabolites identified in wild-type MEFs and Atg7^*−/−*^ MEFs along with the starvation time.

	Metabolite	m/z	Elemental composition	Representative MS/MS ions^b^ (m/z)	*p-*value^c^	Fold change^e^	KEGG	HMDB
Wild-type	Dimethylethanolamine	72.0814	C_4_H_11_NO	57.0583, 72.0817	4.09 × 10^−3^	−6.20	CO4308	HMDB32231
	Dimethylglycine	104.0708	C_4_H_9_NO_2_	60.0818, 104.1076	2.92 × 10^−3^	−2.09	C01026	HMDB00092
	Serine	106.0500	C_3_H_7_NO_3_	60.0454, 70.0661, 88.0765	3.85 × 10^−7^	−8.01	C00065	HMDB00187
	D-(+)-Pyroglutamic acid	130.0497	C_5_H_7_NO_3_	84.0452, 130.0502	2.21 × 10^−3^	−12.92	C02237	HMDB00805
	Aspartic acid	134.0266	C_4_H_7_NO_4_	74.0247, 88.0399, 116.0350	2.33 × 10^−3^	−5.88	C00049	HMDB00191
	Glutamine	147.0760	C_5_H_10_N_2_O_3_	84.0452, 101.0717, 130.0502, 147.0766	1.16 × 10^−4^	−20.50	C00064	HMDB00641
	Phenylacetylglycine	194.0808	C_10_H_11_NO_3_	76.0402, 91.0550, 171.0267	3.66 × 10^−2^	−9.72	C05598	HMDB00821
	Glycerylphosphorylethanolamine	216.0623	C_5_H_14_NO_6_P	136.0484, 216.0650	6.31 × 10^−3^	+3.57	C01233	HMDB00114
	Sphingosine(d18:1)	300.2885	C_18_H_37_NO_2_	89.0609,133.0862,153.0862,252.2683, 282.2793	5.46 × 10^−5^	+3.06	C00319	HMDB00252
	DG(42:9)^d^	357.2597	C_45_H_70_O_5_	264.2697, 330.2358, 357.2614	7.93 × 10^−3^	+6.57	/	HMDB07694
	LysoPE(18:1)^d^	480.3069	C_23_H_46_NO_7_P	339.2902, 480.3072, 497.0568	2.40 × 10^−2^	+4.76	/	HMDB11505
	Threonine^a^	120.0655	C_4_H_9_NO_3_	56.0505, 74.0610, 84.0453, 102.0557, 120.0660	9.31 × 10^−4^	−5.46	C00188	HMDB00167
	Pipecolic acid^a^	130.0860	C_6_H_11_NO_2_	57.0708, 84.0816, 130.0866	3.15 × 10^−3^	−6.06	C00408	HMDB00070
	Acetylcholine^a^	146.1172	C_7_H_16_NO_2_	60.0818,87.0449, 146.1178	3.39 × 10^−2^	−5.51	C01996	HMDB00895
	Lysine^a^	147.1124	C_6_H_14_N_2_O_2_	84.0816, 130.0866, 147.1131	3.11 × 10^−3^	−4.71	C00047	HMDB00182
	Histidine^a^	156.0764	C_6_H_9_N_3_O_2_	95.0612, 110.0719, 156.0771	7.14 × 10^−4^	−6.21	C00135	HMDB00177
	Arginine^a^	175.1185	C_6_H_14_N_4_O_2_	60.0566,70.0661, 116.0711, 130.0978, 175.1193	1.51 × 10^−2^	−7.88	C00062	HMDB00517
	Riboflavin^a^	377.1444	C_17_H_20_N_4_O_6_	153.5018,170.3376,243.0900, 354.9207	7.13 × 10^−3^	−2.99	C00255	HMDB00244
Atg7^*−/−*^	Acetoacetic acid	85.02888	C_4_H_6_O_3_	56.9658, 70.0661, 84.9606	9.05 × 10^−4^	−2.42	C00164	HMDB00060
	Choline	104.1072	C_5_H_12_NO	60.0818, 104.1076	6.56 × 10^−4^	−2.17	C00114	HMDB00097
	Creatine	114.0663	C_4_H_9_N_3_O_2_	72.9381, 90.9485, 114.0668	1.17 × 10^−3^	−2.20	C00300	HMDB00064
	Valine	118.0862	C_5_H_11_NO_2_	55.0553, 72.0817, 118.0867	1.22 × 10^−5^	−3.26	C00183	HMDB00883
	Niacinamide	123.0552	C_6_H_6_N_2_O	79.0551, 105.0341, 123.0557	4.73 × 10^−5^	−4.43	C00153	HMDB01406
	Trans-4-Hydroxy-L-proline	132.0652	C_5_H_9_NO_3_	68.0504, 86.0609, 132.0659	1.93 × 10^−3^	−3.51	C01157	HMDB00725
	Citrulline	176.1026	C_6_H_13_N_3_O_3_	70.0661, 113.0716, 159.0767	2.70 × 10^−3^	−6.38	C00327	HMDB00904
	N6,N6,N6-Trimethyl-L-lysine	189.1592	C_9_H_20_N_2_O_2_	60.0817, 84.0816, 130.0864	3.70 × 10^−3^	+2.27	C03793	HMDB01325
	Myoinositol	203.0519	C_6_H_12_O_6_	112.1127, 203.0530	5.49 × 10^−6^	−2.31	C00137	HMDB00211
	ADMA^d^	203.1497	C_8_H_18_N_4_O_2_	70.0661, 88.0877, 116.0711, 158.1291, 203.1507	2.30 × 10^−3^	−3.88	C03626	HMDB03334
	5-L-Glutamyl-L-alanine	219.0968	C_8_H_14_N_2_O_5_	84.0452, 90.0558, 130.0502, 156.0659, 202.0718	7.79 × 10^−4^	−4.54	C03740	HMDB06248
	5′-CMP	324.0579	C_9_H_14_N_3_O_8_P	97.0292, 112.0512	4.36 × 10^−3^	+2.50	C00055	HMDB00095
	Threonine^a^	120.0655	C_4_H_9_NO_3_	56.0505, 74.0610, 84.0453, 102.0557, 120.0660	1.30 × 10^−5^	−6.50	C00188	HMDB00167
	Pipecolic acid^a^	130.0860	C_6_H_11_NO_2_	57.0708, 84.0816, 130.0866	1.77 × 10^−7^	−10.53	C00408	HMDB00070
	Acetylcholine^a^	146.1172	C_7_H_16_NO_2_	60.0818,87.0449, 146.1178	3.36 × 10^−4^	−3.58	C01996	HMDB00895
	Lysine^a^	147.1124	C_6_H_14_N_2_O_2_	84.0816, 130.0866, 147.1131	2.76 × 10^−7^	−12.10	C00047	HMDB00182
	Histidine^a^	156.0764	C_6_H_9_N_3_O_2_	95.0612, 110.0719, 156.0771	6.05 × 10^−8^	−9.49	C00135	HMDB00177
	Arginine^a^	175.1185	C_6_H_14_N_4_O_2_	60.0566,70.0661, 116.0711, 130.0978, 175.1193	4.35 × 10^−7^	−10.66	C00062	HMDB00517
	Riboflavin^a^	377.1444	C_17_H_20_N_4_O_6_	153.5018,170.3376,243.0900, 354.9207	4.33 × 10^−7^	−5.15	C00255	HMDB00244

^a^Metabolite that can be identified in both wild-type MEFs and Atg7^*−/−*^ MEFs with significant difference.

^b^Metabolites confirmed by chemical standards or by online database and MS/MS fragments.

^c^*p-*values were determined by the ANOVA test.

^d^DG: Diacylglycerol; LysoPE: Lyso-phosphatidyethamine; ADMA: Asymmetric dimethylarginine.

^e^Fold change, representing the maximum fold change along with starvation time, was calculated from the arithmetic mean values of cells under different starvation time. Fold change with a positive value indicates an increasing trend during starvation, while a negative value indicates a decreasing trend.
