# Supplementary material for: Metabolomic Analysis of Mouse Embryonic Fibroblast Cells in Response to Autophagy Induced by Acute Starvation
Source: Sci Rep. 2016 Oct 5;6:34075. doi: 10.1038/srep34075 (PMC5050436; doi:10.1038/srep34075)
Supplement: Supplementary Information [file srep34075-s1.pdf]

Metabolomic Analysis of Mouse Embryonic Fibroblast Cells in Response to Acute Starvation with and without Atg7

Sensen Shen[1], Rui Weng[1, 2], Linnan Li[1], Xinyuan Xu[1], Yu Bai[1]*, Huwei Liu[1]

1. Beijing National Laboratory for Molecular Sciences, Key Laboratory of Bioorganic Chemistry and Molecular Engineering of Ministry of Education, Institute of Analytical Chemistry, College of Chemistry and Molecular Engineering, Peking University, Beijing, 100871
2. Institute of Quality Standard and Testing Technology for Agro-Products, Chinese Academy of Agricultural Sciences, Beijing, 100081

Corresponding Author

* E-mail: yu.bai@pku.edu.cn. Phone: +86-10-62758198. Fax: +86-10-62751708.

Supplementary Table S1. Unidentified altered metabolites in wild-type MEFs

Retention time (min)	m/z	*p*-value *
0.90	131.0530	6.39E-04
0.90	275.0517	3.49E-02
0.91	326.1071	4.07E-02
0.96	104.0708	9.56E-03
1.03	148.0422	1.08E-02
1.29	146.1648	4.05E-02
1.40	203.0789	2.33E-02
1.48	233.9813	4.40E-02
1.82	190.0817	3.67E-03
5.02	188.0676	1.50E-02
5.04	120.0663	1.98E-02
5.29	146.1172	3.39E-02
5.73	159.0913	3.22E-02
6.08	446.4539	2.05E-02
6.08	557.8160	3.49E-02
6.08	743.7534	6.62E-06
6.44	180.1015	3.67E-02
6.95	204.1048	3.50E-02
7.66	305.2576	3.75E-03
8.26	103.0755	2.18E-02
9.12	384.3066	3.05E-02
9.12	449.3697	3.57E-03
9.13	406.3509	5.98E-03
9.14	363.3239	2.91E-02
9.19	493.3960	4.53E-03
9.21	450.3770	4.01E-03
9.73	329.0763	9.12E-07
10.35	119.0816	3.06E-02
10.35	268.1085	1.96E-02
11.21	197.0954	1.20E-04
11.42	457.3153	2.40E-02
12.21	616.4168	2.82E-02

*) *p*-values were determined by one way ANOVA.

Supplementary Table S2. Unidentified altered metabolites in Atg7$^{-/-}$ MEFs.

Retention time (min)	m/z	*p*-value*
0.68	205.4373	8.76E-07
0.73	97.9917	2.92E-03
0.73	263.1454	1.78E-07
0.75	84.0812	5.38E-04
0.76	148.1158	1.52E-06
0.77	220.9339	4.14E-03
0.78	168.0870	1.14E-07
0.80	395.0377	1.28E-04
0.80	259.0635	5.54E-07
0.80	327.0504	7.80E-08
0.80	531.0123	3.64E-07
0.82	190.9114	4.94E-03
0.83	232.9881	9.13E-04
0.84	97.0140	6.68E-04
0.85	200.0401	5.38E-08
0.85	282.9703	6.55E-04
0.85	127.0242	4.51E-07
0.86	76.0399	3.09E-04
0.86	178.0583	9.37E-08
0.86	176.1219	2.84E-07
0.87	191.0398	3.61E-06
0.87	188.9797	1.18E-05
0.87	238.0444	2.15E-03
0.87	105.1105	4.83E-04
0.88	216.0623	3.23E-03
0.88	142.0471	5.41E-05
0.89	383.1145	5.01E-05
0.89	81.0340	6.81E-04
0.91	262.0765	3.55E-07
0.91	203.0175	3.63E-03
0.91	360.0530	3.54E-05
0.96	274.0912	2.88E-06
0.97	178.0705	3.15E-04
0.97	307.0822	4.15E-03
0.97	276.1181	2.53E-06
0.97	346.1475	2.01E-05
0.97	307.0822	4.90E-03
1.03	205.0562	3.97E-03
1.07	150.0579	7.37E-08
1.31	227.0106	4.14E-03
1.47	347.1550	7.47E-05

1.48	205.9866	2.51E-03
1.84	109.0286	7.01E-05
5.03	93.0703	2.94E-06
5.18	380.1474	3.50E-06
5.73	118.0651	4.06E-06
5.73	144.0805	2.86E-04
5.73	159.0913	4.03E-06
5.75	261.1437	2.86E-03
7.36	353.0465	9.47E-05
7.69	273.0899	1.07E-06
9.23	302.2136	1.00E-05
9.54	616.1743	5.67E-08
9.66	273.0899	5.46E-09
9.73	329.0763	1.33E-10
11.21	197.0954	1.12E-04
11.73	279.2307	3.12E-03

*) *p*-values were determined by one way ANOVA

Supplementary Table S3. Summary of the Metabolic Pathway Analysis (MetPA) results.

Pathway category[a]	**Wild-type**	**Atg7$^{-/-}$**
Amino acid metabolism	3[b]. Glycine, serine and threonine metabolism<br>4[b]. Histidine metabolism<br>6[b]. Alanine, aspartate and glutamate metabolism<br>7[b]. Arginine and proline metabolism<br>8[b]. Lysine degradation<br>11[b]. Cysteine and methionine metabolism<br>13[b]. Lysine biosynthesis<br>17[b]. Phenylalanine metabolism	1[c]. Arginine and proline metabolism<br>2[c]. Histidine metabolism<br>6[c]. Lysine degradation<br>7[c]. Glycine, serine and threonine metabolism<br>8[c]. Valine, leucine and isoleucine biosynthesis<br>13[c]. Valine, leucine and isoleucine degradation<br>14[c]. Lysine biosynthesis<br>22[c]. Tyrosine metabolism
Energy metabolism	1[b]. Nitrogen metabolism<br>16[b]. Methane metabolism	9[c]. Nitrogen metabolism
Carbohydrate metabolism		4[c]. Ascorbate and aldarate metabolism<br>16[c]. Butanoate metabolism<br>18[c]. Inositol phosphate metabolism<br>21[c]. Galactose metabolism
Metabolism of cofactors and vitamins	10[b]. Riboflavin metabolism<br>14[b]. Biotin metabolism	10[c]. Riboflavin metabolism<br>11[c]. Pantothenate and CoA biosynthesis<br>12[c]. Nicotinate and nicotinamide metabolism<br>15[c]. Biotin metabolism
Lipid metabolism	9[b]. Sphingolipid metabolism<br>12[b]. Glycerophospholipid metabolism	5[c]. Synthesis and degradation of ketone bodies<br>19[c]. Glycerophospholipid metabolism

Metabolism of other amino acids	2 [b]. D-glutamine and D-glutamate metabolism<br>15 [b]. Cyanoamino acid metabolism	17 [c]. Glutathione metabolism
Nucleotide metabolism	18 [b]. Pyrimidine metabolism<br>19 [b]. Purine metabolism	20 [c].Pyrimidine metabolism
Translation	5 [b]. Aminoacyl-tRNA biosynthesis	3 [c]. Aminoacyl-tRNA biosynthesis

a) Pathway category refers to the KEGG-based database pathway.

b) Numbers were corresponding to the number listed in Supplementary Figure S1.

c) Numbers were corresponding to the number listed in Supplementary Figure S2.

Supplementary Table S4. The primers used for real-time quantitative PCR (qPCR).

Primer designation	Primer sequence(5'to3')
Sphk1-f	ACTCCACCTTTGGAGGTTGC
Sphk1-r	GCGGCCAGATTTTTAGCTTCC
Sgpp2-f	CCTGCGGGATTCACAGCTC
Sgpp2-r	TCCTCTCCAGAAGCCTCCC
Sgpl1-f	CGTGAGACGCAGAGGCAG
Sgpl1-r	GCTCGAAGTCCTTCAGCTTG
Sptlc2-f	CCAAAATTGGCGCCTTTGGA
Sptlc2-r	GGGTAGCAGGAAATCCCACC
Sds-f	CTCTGTCCAGCTCCTCCTGT
Sds-r	GCCTTGTTTTGCTTTCATCTTGC
Shmt2-f	TTCACTCGAACTTCACGGGG
Shmt2-r	AGCTGACCACATCTCCGAGT
Gls-f	ACGTCAGATGGTGTCATGCT
Gls-r	CAGCAACCTTCCCTCCAGAC
Pdha1-f	GTTTTGGGCGTGGCTTCG
Pdha1-r	CGGCTTGCCGGCTTCT
ACTB-f	CACTGTCGAGTCGCGTCC
ACTB-r	TCATCCATGGCGAACTGGTG

Supplementary Table S5. The definition and functions of the selected proteins based on NCBI.

Gene name	Definition	Functions of its encoding protein	NCBI ID
Sphk1	Sphingosine kinase 1	An enzyme that phosphorylates sphingosine into sphingosine-1-phosphate	20698
Sgpp2	Sphingosine-1-phosphate phisphotase 2	An intracellular enzyme located in the endoplasmic reticulum, which regulates the level of sphingosine-1-phosphate (S1P)	43323
Sgpl1	Sphingosine phosphate lyase 1	An enzyme which catalyzes the S1P into free fatty acids or phosphate lipids	20397
Sptlc2	Serine palmitoyltransferase	The key enzyme in sphingolipid biosynthesis which catalyzes the pyridoxal-5-prime-phosphate-dependent condensation of L-serine and palmitoyl-CoA to 3-oxosphinganine	20773
Sds	Serine dehydratase	An enzyme that catalyzes the conversion of L- , D-serine, or L-threonine to pyruvate/ketobutyrate	231691
Shmt2	Serine hydroxymethyltransferase2	An enzyme which carries out interconversion of serine and glycine	108037
Gls	Glutaminase	An enzyme that deaminates glutamine to glutamate	14660
Pdha1	Pyruvate dehydrogenase E1 alpha 1	An enzyme which catalyzes the irreversible oxidative decarboxylation of pyruvate to produce acetyl-CoA in the bridging step between glycolysis and the citric acid cycle	18597

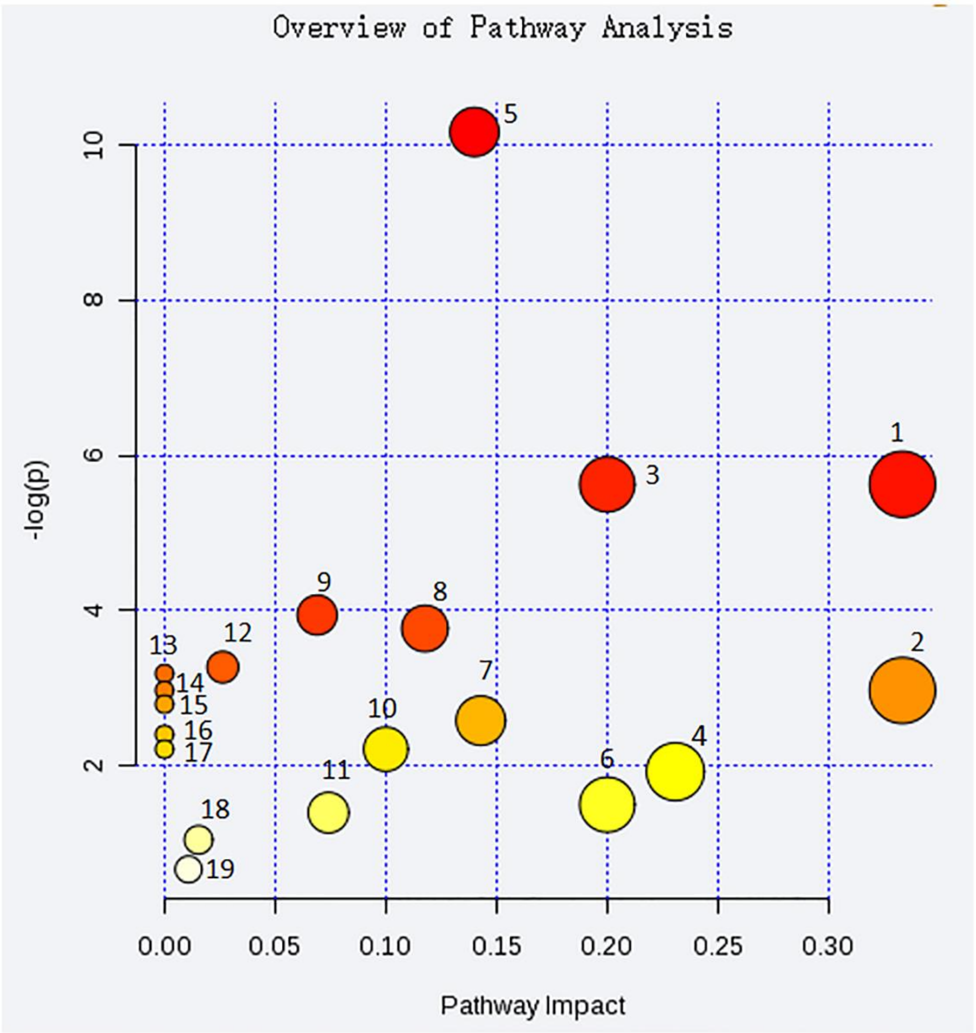

Supplementary Figure S1. Pathway analysis of the altered metabolites in wild-type MEFs using MetPA. The dots mean the affected pathways along with the starvation time, with each number corresponding to the number listed in Supplementary Table S1. Pathways were arranged according to enrichment analysis (y axis) and topology analysis (x axis) scores (see "Materials and methods" section and Supplementary Table S1 for details).

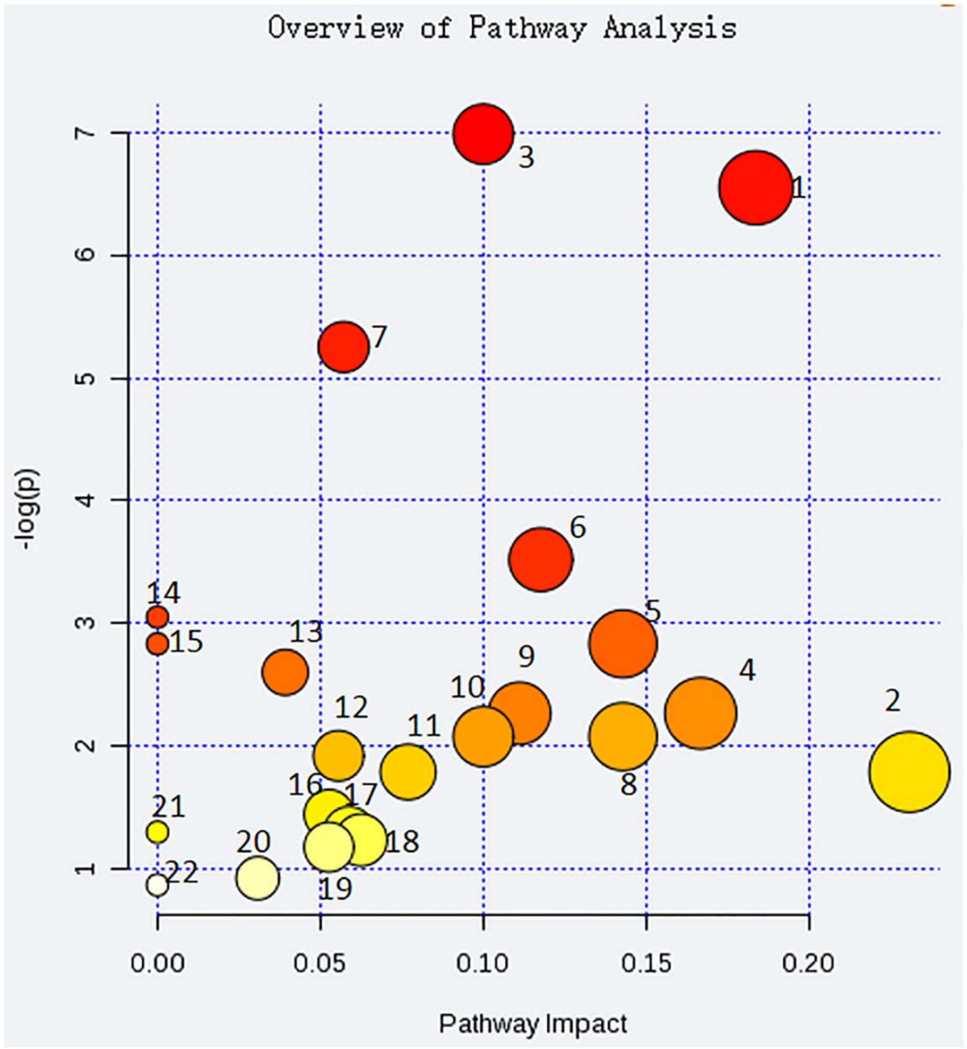

Supplementary Figure S2. Pathway analysis of the altered metabolites in Atg7$^{-/-}$ MEFs using MetPA. The dots mean the affected pathways along with the starvation time, with each number corresponding to the number listed in Supplementary Table S1. Pathways were arranged according to enrichment analysis (y axis) and topology analysis (x axis) scores (see "Materials and methods" section and Supplementary Table S1 for details).

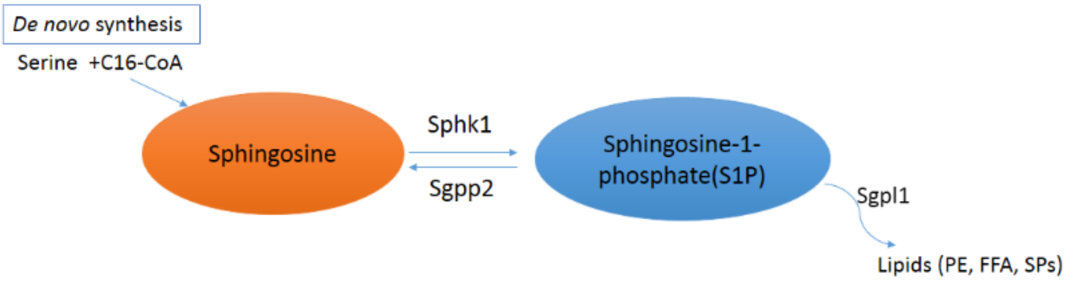

Supplementary Figure S3. Mutual transformation between sphingosine and sphingosine-1-p (S1P) and the related enzymes. Sphk1: sphingosine kinase 1; Sgpp2: sphingosine -1-phosphate phosphatase 2; Sgpl1: sphingosine phosphate lyase 1.

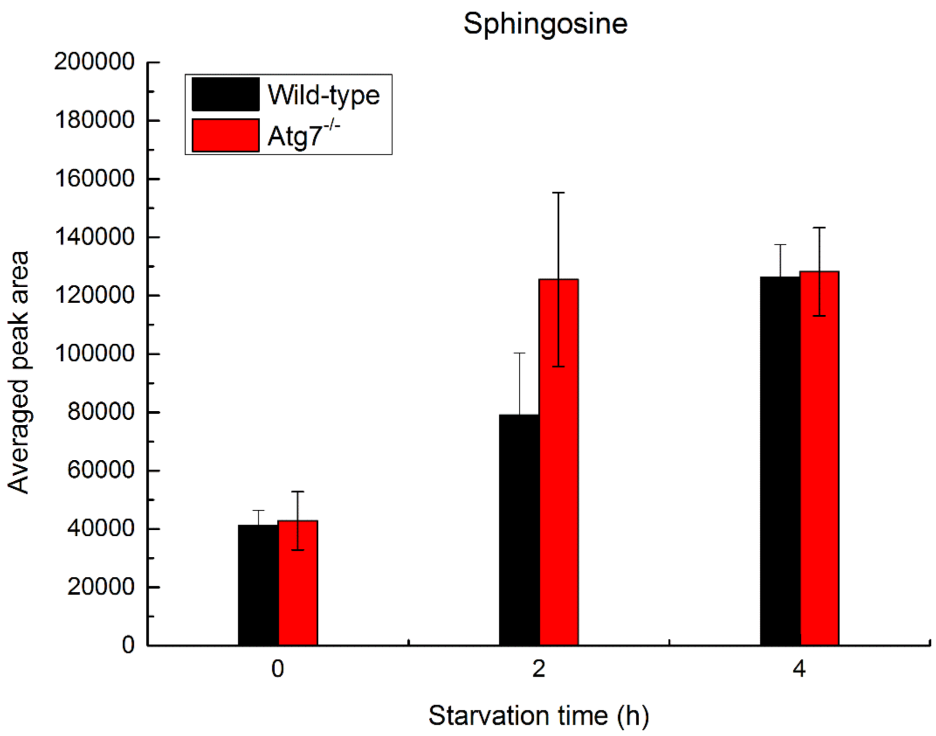

Supplementary Figure S4. The tendency of sphingosine (d18:1) along with the starvation time. Wild-type MEFs and $Atg7^{-/-}$ MEFs were treated with EBSS for the indicated times. Error bars indicate the SD.

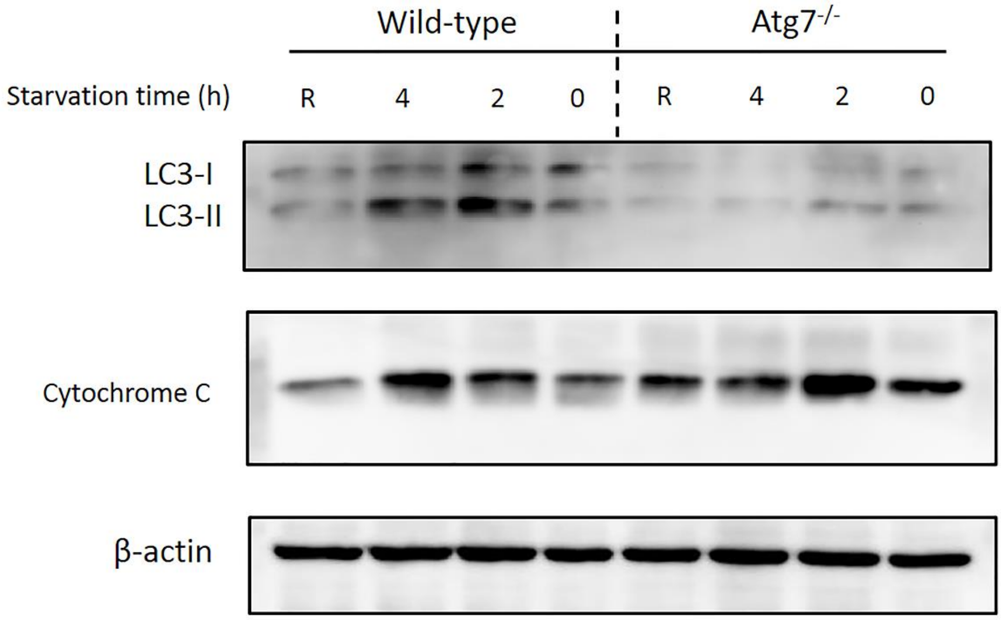

Supplementary Figure S5. Western blot results of the conversion of LC3-I to LC3-II and Cytochrome C in wild-type and Atg7$^{-/-}$ MEFs. Cells were treated with EBSS for the indicated times. R: recovery group, in which cells were re-cultured in the full medium after 4 hours' starvation.

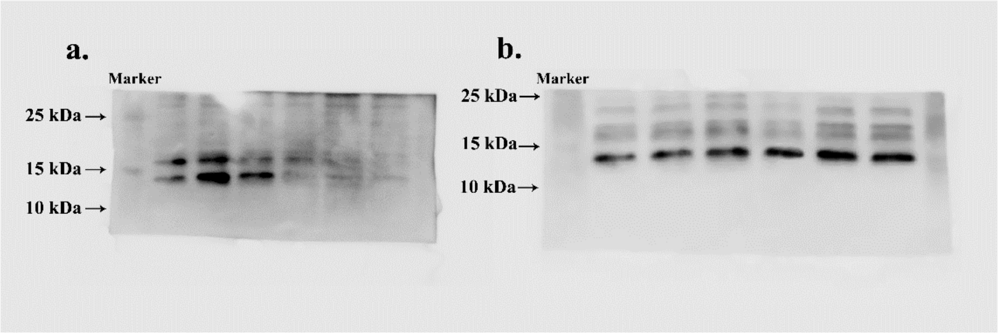

Supplementary Figure S6. Full-length western blotting assay a) Conversion of LC3-I to LC3-II. **b)** Level of cytochrome C.
